# A simple, reproducible method for monitoring the treatment of tumours using dynamic contrast-enhanced MR imaging

**DOI:** 10.1038/sj.bjc.6603140

**Published:** 2006-05-02

**Authors:** B Morgan, J F Utting, A Higginson, A L Thomas, W P Steward, M A Horsfield

**Affiliations:** 1 Department of Cancer Studies and Molecular Medicine, University of Leicester, Leicester Royal Infirmary, Leicester LE1 5WW, UK; 2 Department of Medical Physics, University Hospitals of Leicester NHS Trust, Leicester LE1 5WW, UK; 3 Department of Radiology, University Hospitals of Leicester NHS Trust, Leicester LE1 5WW, UK; 4 Department of Cardiovascular Sciences, University of Leicester, Leicester Royal Infirmary, Leicester LE1 5WW, UK

**Keywords:** dynamic contrast-enhanced magnetic resonance imaging, DCE–MRI, biomarker, clinical trial

## Abstract

Dynamic contrast-enhanced MR imaging (DCE-MRI) may act as a biomarker for successful cancer therapy. Simple, reproducible techniques may widen this application. This paper demonstrates a single slice imaging technique. The image acquisition is performed in less than 500 ms making it relatively insensitive to respiratory motion. Data from phantom studies and a reproducibility study in solid human tumours are presented. The reproducibility study showed a coefficient of variation (CoV) of 19.1% for *K*^trans^ and 15.8% for the initial area under the contrast enhancement curve (IAUC). This was improved to 16 and 13.9% if tumours of diameter less than 3 cm were excluded. The individual repeatability (the range within which individual measurements are expected to fall) was 30.6% for *K*^trans^ and 26.5% for IAUC for tumours greater than 3 cm diameter. This approach to DCE–MRI image acquisition can be performed with standard clinical scanners, and data analysis is straightforward. For treatment trials with 10 patients in a cohort, the CoV implies that the method would be sensitive to a treatment effect of greater than 18%. The individual repeatability is well inside the 40% change shown to be important in clinical studies using this DCE–MRI technique.

Dynamic contrast-enhanced magnetic resonance imaging (DCE–MRI) is the continuous acquisition of MR images over a period of between 1 and 20 min after the injection of a contrast agent into a peripheral vein, typically in the antecubital fossa. Normally, this involves a gadolinium chelate, which causes an increase in signal intensity on *T*_1_-weighted MR images. The increase in signal intensity (enhancement) is caused by the change in the nuclear spin relaxation rate *R*_1_ (*R*_1_=1/*T*_1_), which is proportional to the concentration of the contrast agent. Enhancement, as measured by DCE–MRI, may be used to study the pathophysiology of tumours (Taylor *et al*, 1999; Yuh, 1999; Morgan *et al*, 2004). Parameters of interest include tumour vascularity, vascular endothelial permeability and the volume fraction of the extravascular, extracellular space (Knopp *et al*, 1999). There is an increasing interest in the use of these techniques as biomarkers in the development of novel cancer therapies (Jayson *et al*, 2002; Galbraith *et al*, 2003; Morgan *et al*, 2003; Leach *et al*, 2005; Rehman and Jayson, 2005; Thomas *et al*, 2005). Different approaches to the acquisition and analysis of DCE–MRI studies have been reviewed elsewhere (Morgan *et al*, 2004; Buckley and Parker, 2005; Parker and Buckley, 2005).

Ideally, DCE–MRI data acquisition should have high spatial resolution allowing mapping of enhancement parameters for the tumour in a pixel-by-pixel manner. This is important for gaining prognostic information about a tumour where small areas of increased tumour activity could otherwise be missed (Degani *et al*, 1997; Parker *et al*, 1997). This approach typically involves image acquisition times of between 6 and 30 s for each image in the dynamic series. As a large percentage of metastatic disease treated by chemotherapy is in parts of the body that cannot be easily immobilised, such as the liver and lung, pixel-by-pixel data analysis is complicated by the need for sophisticated registration of the tumour in consecutive images. Long imaging times also involve multiple breath holds, which may be difficult for a patient with advanced cancer. This suggests an approach with short image acquisition times that can freeze motion and an analysis based on the whole tumour as the region of interest. A rapid imaging approach may also allow better depiction of the early part of the contrast enhancement curve. Successful systemic treatment may be expected to have an effect detectable by averaging the signal from the whole region of interest (ROI), but changes that are restricted to a small part of the tumour, or to poorly enhancing pixels, could be missed.

One problem when analysing tumour microcirculation is that signal intensities obtained from the MRI scanner are arbitrary and need to be ‘standardised’ if comparisons between different patients or patient visits are required. One possible method is by dividing the signal change due to gadolinium enhancement by the precontrast signal of the tumour (Mayr *et al*, 1999; Reddick *et al*, 1999). This has the disadvantage that the precontrast *R*_1_ can change during therapy, for example with increasing tumour oedema, causing an apparent change in enhancement (Evelhoch, 1999). A mechanism to standardise signal intensities is therefore required that is not sensitive to changes in pre-contrast *R*_1_.

We have shown that high temporal resolution DCE–MRI, using a single slice approach with a single ROI evaluating the whole tumour cross-section, performed without breath holding, is feasible and can act as a biomarker for the beneficial effects of an anti-angiogenesis agent (Morgan *et al*, 2003; Thomas *et al*, 2005). However, in order to plan such trials it is important to know the inherent variability of the results from the DCE–MRI approach in order to assess the significance of individual changes, and to use power statistics to determine the cohort sizes required to give statistical significance for a desired effect.

When planning a multicentre clinical trial for patients with advanced cancer, a DCE–MRI technique that is quick, does not involve multiple breath holds and is applicable in all areas of the body has obvious advantages. A validated, straightforward way of analysing the data from DCE–MRI would allow wider application of the technique. This study shows the rationale for developing a single slice DCE–MRI technique and investigates different approaches to standardising the measured signal intensities, and analysing the time course of signal intensities from an ROI that covers the whole tumour cross-section. The chosen imaging sequence was performed in cancer patients on two occasions 1 week apart, without treatment, in order to assess the reproducibility of several common approaches used for analysing DCE–MRI data.

The results of this reproducibility analysis were then put in a clinical context by re-analysing and comparing the data obtained in a previous published clinical trial that successfully used this technique as a biomarker (Thomas *et al*, 2005). In this dose escalating phase I study, the pharmacodynamic effect of PTK787/ZK222584 (PTK/ZK), an orally active inhibitor of vascular endothelial growth factor (VEGF) receptor tyrosine kinases, which inhibits VEGF-mediated angiogenesis, was evaluated.

## MATERIALS AND METHODS

### Pulse sequence

To achieve high temporal resolution and avoid the need for breath holding, we used scan parameters with individual image acquisition times of less than 500 ms, effectively freezing motion owing to respiration. To achieve this, a repetition time (TR) of 5 ms or less is necessary for 100 phase encoding steps. *T*_1_ weighting can then be created by applying a magnetisation preparation pulse (Snapshot FLASH) (Haase, 1990). We used an inversion pulse for magnetisation preparation; this pulse was nonslice selective (i.e. affecting the whole bore of the magnet) to minimise the effects of through-plane motion and blood inflow. As respiratory motion in the abdomen and lower part of the lung is largely in the cranio–caudal direction, a coronal or sagittal oblique plane was used to keep the tumour within the imaged slice during the dynamic run. The parameters of the sequence used were: TR=3.3 ms, echo time (TE)=1.4 ms, flip angle (*α*)=8°, *k* space matrix 100 phase encoding steps × 128 points in the readout direction, inversion time (TI)=655 ms, and time between successive inversion pulses (TR_0_)=3000 ms.

### Quantification of *R*_1_

Assuming minimal transverse relaxation between the excitation pulses and signal acquisition, the contrast obtained from an inversion recovery snapshot FLASH sequence is affected by TR, TI, *α*, the number of lines of *k* space (*Nk*) and the delay between the end of image acquisition and the next inversion pulse (TD). From previous work (Jivan *et al*, 1997), the *z* magnetisation after *m* phase encoding steps relative to the equilibrium magnetisation (*M*_0_) is given by: 
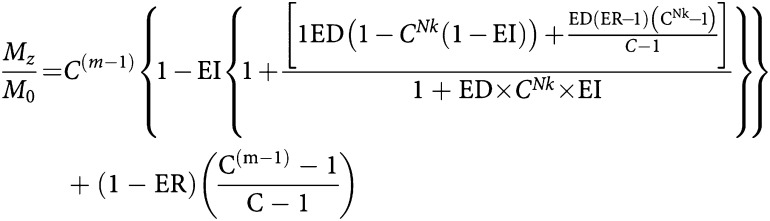
 where 
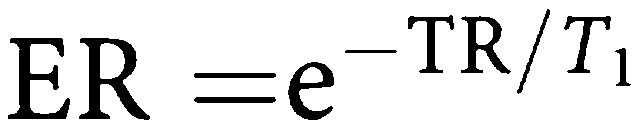
, 
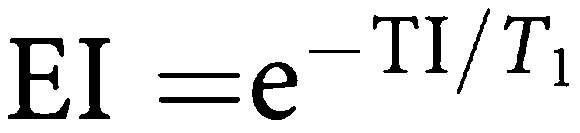
, 
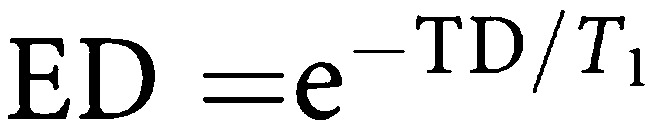
 and C=ER cos *α*.

The sum of TI, TD and (TR × *Nk*) is TR_0_, the time between successive inversion pulses. As TR_0_ → *∞* and *α* → 0, then equation (1) simplifies to 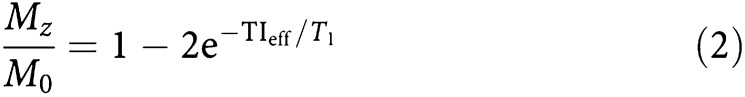 where TI_eff_ is the time from the inversion pulse to the centre of *k* space. The signal intensity is given by *S*=*M*_*z*_ sin *α*.

In order to provide data that are comparable between patient visits, it is necessary to convert signal intensities to quantitative data. One method is to calculate the relaxation rate *R*_1_, which can be done by acquiring an image without the inversion pulse applied, after the contrast enhanced run, removing a major component of *T*_1_ weighting. The ratio of signal intensities with (*S*) and without (*S*_0_) the inversion pulse is then related to the *R*_1_ of the tissue by equations (1) and (2), from which *R*_1_ may be determined.

### Phantom study

This approach to *R*_1_ estimation was tested in a phantom study. The phantom consisted of vials of water doped with different concentrations of Gd-DTPA giving a range of *R*_1_ values from 0.4 to 10 s^−1^. The *R*_1_ values were accurately measured using an IR-prepared turbo spin echo (TSE) sequence with TE=10 ms, TR=5000 ms, echo train length=5, 128 × 128 matrix, 1.56 mm in plane resolution, 5 mm slice thickness and inversion times of 23, 123, 323, 723 and 1520 ms. Phantom work was carried out using a Siemens symphony 1.5 T system (Siemens, Erlangen, Germany). Based on equation (1) this sequence effectively nulls signal at an *R*_1_ of 0.53 s^−1^. Where *R*_1_ was less than 0.53 s^−1^, the positive signal of the magnitude images was inverted. In a DCE–MRI study, rectified signal can be recognised if the signal reduces in the first few images of contrast enhancement. The estimated *R*_1_ values, using the chosen DCE–MRI sequence and equations. (1) and (2), were compared with the accurately measured *R*_1_ values.

### Clinical study

Eleven patients with advanced cancers, including colorectal cancers with liver metastases, were recruited as part of two ‘phase I’ trials. Patients had two DCE–MRI scans 1 week apart, without treatment. All patients were informed about the investigational nature of the study according to institutional and regional guidelines, and subsequently gave informed consent before start of the study. Permission of local ethics regulatory bodies was obtained.

All MR images were acquired using a 1.5 T whole body magnet equipped with 25 mTm^−1^ gradient coils and phased array surface coils (Siemens Magnetom Vision, Erlangen, Germany). All patients underwent standard transverse breath hold gradient recalled echo (GRE) *T*_1_ and TSE *T*_2_-weighted imaging of the tumour region (GRE, typically TR=150 ms, TE=4 ms, *α*=70° and matrix 256 × 256; TSE typically TR=5000 ms, TE=90, *α*=90°, echo train length=23). In regions where respiratory motion was a factor, the *T*_1_-weighted multislice GRE examination was repeated during gentle respiration to improve slice positioning for the dynamic series, which was also performed during gentle respiration. A representative disease site was selected (target lesion) and a single coronal oblique slice was planned to bisect the midline of the tumour and a major blood vessel (usually aorta). This became the imaged tumour for all subsequent scans. The DCE–MRI sequence was then run with 100 measurements, 3 s apart (TR_0_=3000 ms). Low molecular weight gadolinium-chelate 0.1 mmol kg^−1^ (Magnevist, Schering AG or Omniscan, Nycomed) was injected as a rapid bolus through an arm vein in less than 5 s by hand injection (a power injector was not available at the time of the study). This injection speed was adhered to for all patients. Both contrast agents display comparable relaxivity and distribution characteristics and therefore would be expected to provide similar enhancement parameters (Sze *et al*, 1991). The same contrast agent was always used for individual patients. Injection commenced after the first four measurements to allow magnetisation to reach a steady state. At the end of the dynamic run, the sequence was repeated with the inversion pulse switched off to acquire the *S*_0_ image. For subsequent scans, patient positioning and slice orientation was replicated using the previous image set.

Signal intensities were taken from regions of interest drawn around the tumour and a major blood vessel (e.g. aorta) separately using an image analysis package, Analyze™ (Mayo Clinic, Rochester, MN, USA). For the tumour, the ROI was drawn based on the precontrast images after review of the contrast-enhanced images so that large blood vessels and non-tumour tissues were avoided. For the major blood vessel, the ROI was drawn based on the peak arterial enhancement phase of imaging. The positions of the ROIs were corrected on a time point by time point basis for any movement during the dynamic run. The shapes of the ROIs were not changed throughout the dynamic run. The maximum diameter and appearance on *T*_1_- and *T*_2_-weighted images of the target lesion was recorded. The slice position for the dynamic run was checked on both *T*_1_- and *T*_2_-weighted images to ensure consistency between scans and care was taken to ensure that the ROIs had the same anatomical positions and sizes for both scans.

In clinical DCE–MRI, measurement of *S*_0_ at every time point would result in unacceptably low temporal resolution. Therefore, we performed a single measurement of *S*_0_ at the end of the dynamic run, by acquiring an image without the inversion preparation pulse. Equation (1) shows that measured *S*_0_ depends on *R*_1_ as there is still some *T*_1_ weighting, owing to spin relaxation during the TR intervals, even when TI → ∞ (the equivalent of turning the inversion pulse off). A single reading of *S*_0_ therefore produces inaccurate estimates of *R*_1_ when *R*_1_ differs from its value at the time when *S*_0_ is measured. An estimate of the maximum error introduced by the assumption of a constant *S*_0_ was made using estimates of *R*_1_ precontrast and at maximum enhancement. Equation (1) was used to predict the true *S*_0_ at each of these time points. *R*_1_ calculations were then performed using equation (2), firstly with the single measured *S*_0_ value and second using the true *S*_0_ values (corrected for *R*_1_). This error analysis was performed for typical enhancement curves seen in the clinical study.

The signal intensities were preprocessed in three forms as summarised in Table 1. First, the raw signal intensities were used unchanged. In this form, any variability caused by different coil sensitivity or receiver gain on the two visits, or by different contrast bolus characteristics remained uncorrected. Secondly, the signal intensities were divided by the initial area under the arterial enhancement curve (IAUC-A) for a defined period of time. The IAUC-A was calculated by first subtracting the average pre-contrast signal intensities in the artery from the arterial time series, then using the trapezium rule to calculate the total area under the resulting change in enhancement out to times of 60 and 180 s (IAUC-A(60) and IAUC-A(180)) after contrast injection. Thirdly, the signal intensities were converted to *R*_1_ values using equation 2) and the *S*_0_ image collected after the end of the dynamic run.

**Figure 1:**
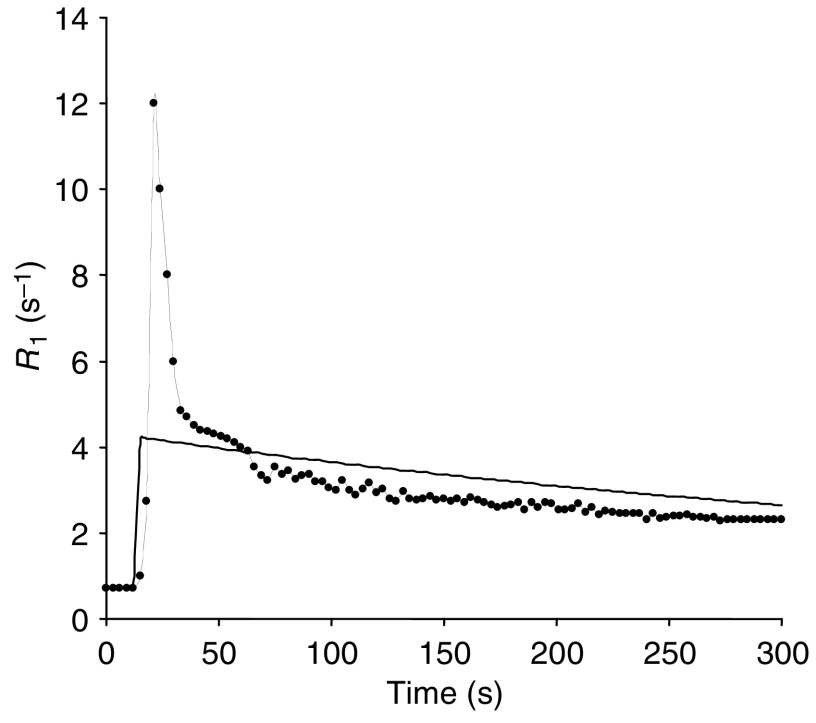
Change in *R*_1_ against time used to represent a standard AIF for a 0.1 mmol kg^−1^ gadolinium chelate injected as a bolus. The continuous line represents the AIF calculated using a formula from a model (Tofts and Kermode, 1991). The dotted line is our standard AIF created from locally acquired data, and modified based on published data (Fritz-Hansen *et al*, 1996) for high concentrations obtained in the early part of the enhancement curve owing to the contrast bolus.

These three data sets were then used to calculate peak enhancement and the initial area under the enhancement curve (calculated as above) for 60 and 180 s (IAUC(60) and IAUC(180)). The bidirectional transfer constant *K*^trans^ was also calculated using a two compartment model (Tofts *et al*, 1999). As the imaging parameters are optimised for tumour rather than arterial enhancement, it was not possible to calculate *R*_1_ accurately for the arterial ROI. *K*^trans^ was therefore measured in two ways: first using the raw signal intensities from the tumour and arterial ROIs, and secondly using the *R*_1_ values from the tumour, and a standard data set for aortic input, similar to previous published data (Figure 1) (Tofts and Kermode, 1991; Fritz–Hansen *et al*, 1996). The timing of the start of this standard arterial input function (AIF) was measured from the artery imaged in the study.

The data obtained in a previous published clinical trial that successfully used this technique as a biomarker (Thomas *et al*, 2005) were re-evaluated. Dynamic contrast-enhanced MR imaging was performed pre-treatment, on day 2, and at the end of the 28-day cycle to show changes in the contrast enhancement parameters of tumours in 35 patients with advanced cancer. *K*^trans^, IAUC(60) and IAUC(180) were calculated. These data are presented as per cent change from the pretreatment value after treatment and are then compared with pharmacokinetic end points.

### Statistical analysis

Dynamic contrast-enhanced MR imaging is applied to assess changes caused by treatment, with patients acting as their own controls. Previous experience shows a wide interpatient coefficient of variation (CoV) of 61% in pretreatment values for *K*_i_ (Morgan *et al*, 2003), equivalent to *K*^trans^ (Tofts *et al*, 1999). This considerable variability in the pre-treatment enhancement of tumours makes the percentage rather than absolute changes of enhancement parameters more amenable to analysis. Statistical analysis was therefore performed on the percentage changes of the observed parameter between the two scans. The percentage change data were tested for normality of distribution by the Shapiro–Wilk test. The mean change and CoV were calculated and expressed as percentages (Bland and Altman, 1996a). Assuming that the post-treatment variability is similar, the CoV can be used to assess the statistical power of studies, or anticipate the patient numbers required for a study to demonstrate a given degree of treatment effect. The repeatability value was also evaluated (Bland and Altman, 1996a), which is defined as the range within which 95% of measurements will fall, assuming no treatment effect, and is therefore helpful in assessing the significance of individual patient results.

## RESULTS

### Pulse sequence, quantification of *R*_1_ and phantom study

**Figure 2:**
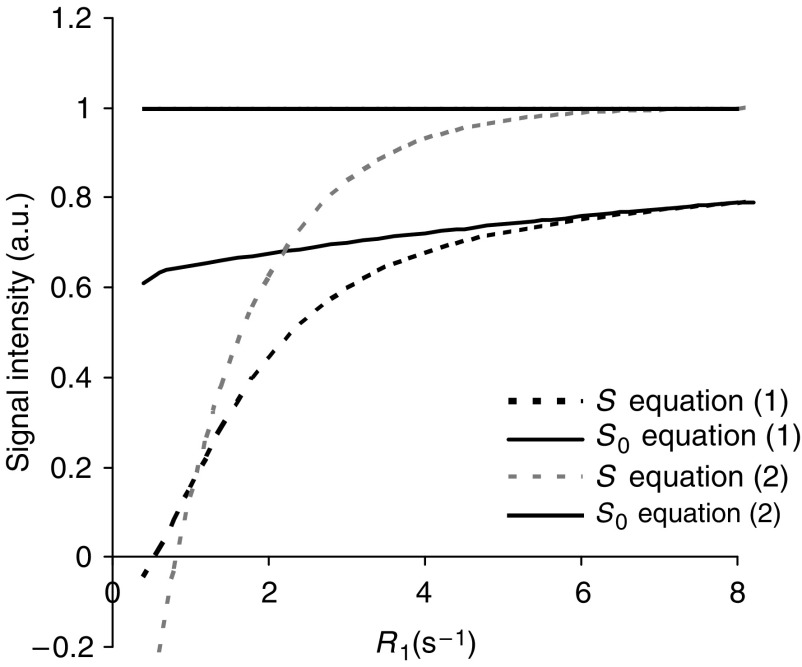
Signal intensities for the IR snapshot FLASH sequence predicted using equation (1) and the simplified equation (2). TR=3.3 ms, TE=1.4 ms, *α*=8°, TI=655 ms, TR_0_=3000 ms.

**Figure 3:**
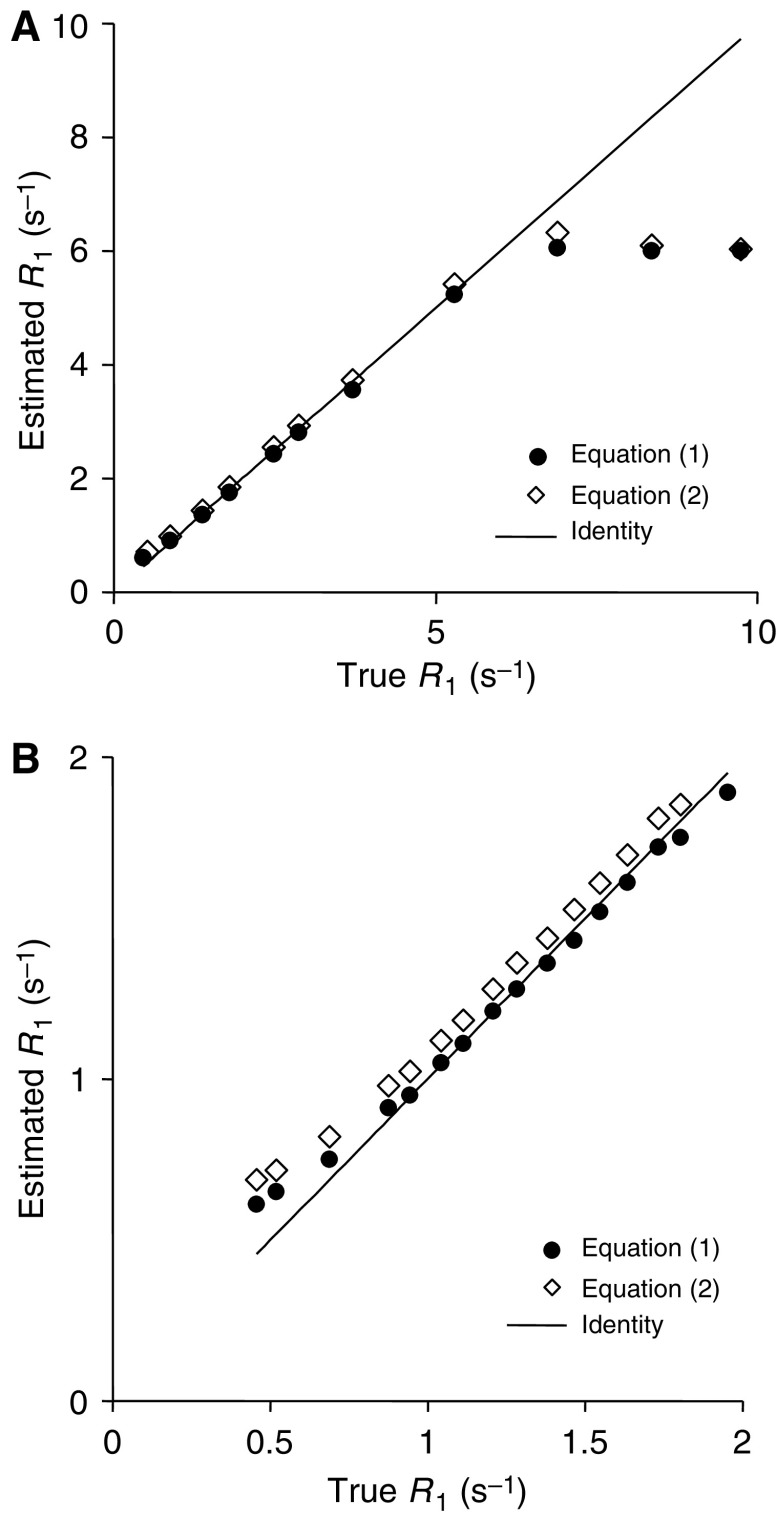
Comparison of *R*_1_ estimated from the DCE–MRI sequence using equations (1) and (2) and the ‘true’ value measured by IR-prepared turbo spin echo sequence in a phantom with *R*_1_=0.5–10 s^−1^ (**A**) and 0.5–2 s^−1^ (**B**).

Figure 2 shows simulated signal intensities for the IR snapshot FLASH sequence, using equation (1) and the simplified equation (2) for the sequence described above. Although there is considerable difference between the signal intensities predicted by the two expressions, Figure 3 shows that *R*_1_ can be estimated accurately for *R*_1_ values between 1 and 5 s^−1^ using either equations (1) and (2). The marked error occurring at *R*_1_ values above 5 s^−1^ is due to almost complete relaxation during the TI interval when *S* approaches *S*_0_, as shown in Figure 2. Thus, with this sequence, selection of sequence parameters to accurately measure relatively short *R*_1_ compromises the ability to measure long *R*_1_ values accurately. The chosen TI_eff_ is therefore a compromise based on the pre-contrast and range of *R*_1_ seen in tumours during DCE–MRI.

### Clinical study

**Figure 4:**
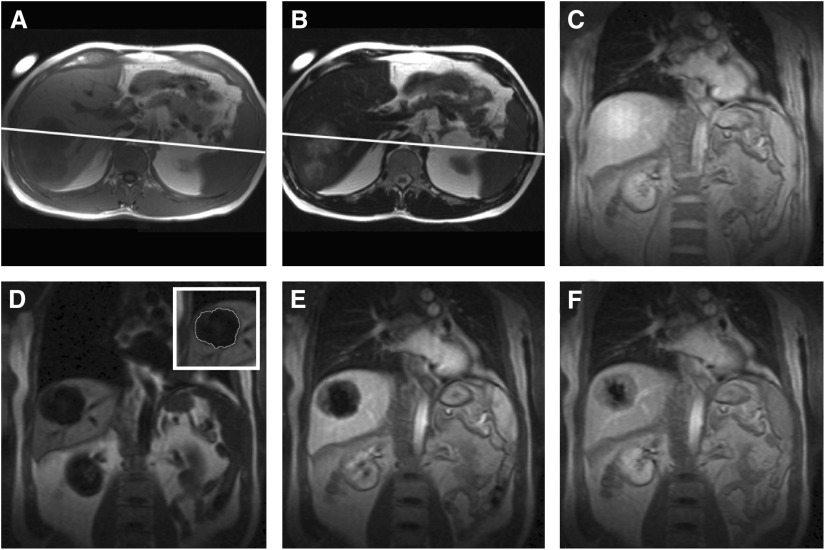
Image set showing a *T*_1_ (**A**) and *T*_2_ (**B**)-weighted image, demonstrating the tumour and the planned dynamic slice position (white line). Image (**C**) is the post contrast *S*_0_ image. The dynamic contrast enhancement series is represented by a precontrast (**D**), and early and late contrast enhanced images (**E** and **F**). The inset image in (**D**) shows how the ROI is drawn for analysis.

Of the 11 patients recruited, one patient was excluded owing to incorrect positioning of the imaging slice on the second scan. Despite breathing motion, it was possible to maintain the size and shape of the tumour and arterial ROI in all remaining cases. Figure 4 demonstrates an example of the images obtained for a liver metastasis. Although all slices included a major artery (aorta or iliac artery), ghosting from motion artefact in the phase-encoded direction was not a significant problem, presumably due to rapid image acquisition and the nonselective inversion pulse. The minimum observed precontrast *R*_1_, averaged over the whole ROI, was 0.95 s^−1^ (*T*_1_=1.05 s). The maximum *R*_1_ was 3.4 s^−1^ (*T*_1_=0.3 s).

**Figure 5:**
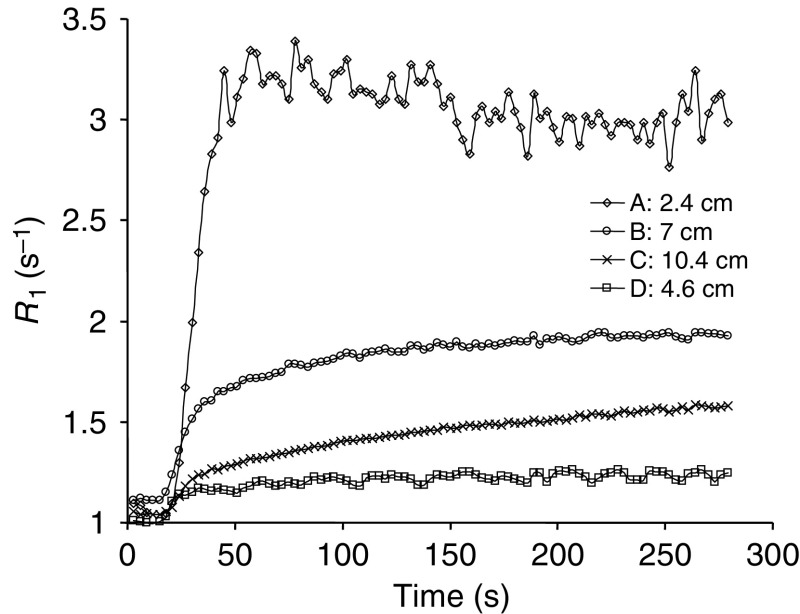
The *R*_1_ time courses for four DCE–MRI series, covering the range of contrast enhancement and tumour size. The highest variation in *R*_1_ between data points is seen for tumour A, which is the smallest measured tumour. Although the smoothness of the enhancement curve is related to tumour size, all cases are amenable to analysis.

From Figure 5, it can be seen that the smoothness of the curves improves with increasing tumour size. As *S*_0_ is not continuously measured as *R*_1_ changes, errors are introduced in the dynamic measurement of *R*_1_, which can be estimated as described earlier. For lesion A (precontrast *R*_1_=1 s^−1^, maximum *R*_1_=3.4 s^−1^, and *R*_1_ at *S*_0_ signal acquisition=3 s^−1^), the error in the maximum change of estimated *R*_1_ (2.4 s^−1^) is 0.13 s^−1^ or 5.4%. For lesion B, there is gradually increasing enhancement throughout (precontrast *R*_1_=1 s^−1^, maximum *R*_1_∼2 s^−1^) and the error in the maximum change of estimated *R*_1_ (1 s^−1^) is approximately 0.009 s^−1^, or less than 1%. For lesions C and D, the maximum estimated error is also less than 1%. As tissue contrast enhancement changes slowly at the end of the dynamic sequence (Figure 5), and the *S*_0_ sequence is only mildly sensitive to these changes, the signal intensity in *S*_0_ image will change very little in the period from the end of the dynamic image sequence to the acquisition of the *S*_0_ image.

In this study, there was correlation between the difference in the enhancement parameters of repeated measurements in a single patient and their mean value for that patient (for *K*^trans^ *r*=0.42, Pearson's correlation coefficient). This causes a skew of the ‘normal’ distribution and a transformation of the data should be performed in these circumstances (Bland and Altman, 1996b, 1996c). As stated in the Materials and Methods section, the data were transformed to percentage difference from the first scan to the second. There was no significant correlation between the percentage difference and the mean measured parameters and therefore no further data transformation was required. For all listed parameters expressed as percentage change, there was no significant evidence against a normal distribution (Shapiro–Wilk test). The mean difference, CoV and repeatability are shown for all measured parameters in Table 1.

In this study, use of raw signal intensities for calculating peak enhancement and IAUC resulted in high CoV, which is improved by dividing by the arterial IAUC. Even though the peak enhancement is taken from just a single data value along the enhancement curves, rather than an integration of many more data points as is the case with IAUC, the reproducibility is very similar to IAUC. This is a reflection of the smoothness of the tumour enhancement curves. The *K*^trans^ calculated from tumour and arterial signal intensities showed a higher CoV (34%) than the *K*^trans^ from tumour *R*_1_ and a standard AIF (19.1%). This is probably a reflection of the fact that any real variation in the AIF between the two scans is outweighed by the fact that the AIF is measured inaccurately with this sequence.

The individual patient data for two commonly used parameters, *K*^trans^ and IAUC[60], calculated from *R*_1_ values, are given in Table 2. Although no correlation was seen between *T*_2_ signal intensity and enhancement parameters, the second case in Table 2 had very high *T*_2_ compared with the other cases, consistent with a cystic nature of the metastasis. Guidelines from a recent US national cancer institute workshop on DCE–MRI state that tumours in a fixed superficial location should be at least 2 cm in diameter and other tumours should be at 3 cm in diameter (2004). This study shows a tendency for greater variability with reducing size, and excluding lesions less that 3 cm in diameter reduced CoV. The colorectal liver metastases group also had lower CoV and repeatability values (*K*^trans^ 14.2 and 26.5% and IAUC(60) 11 and 21.3%, respectively), although this may be related to the fact that this group had relatively larger tumours.

**Figure 6:**
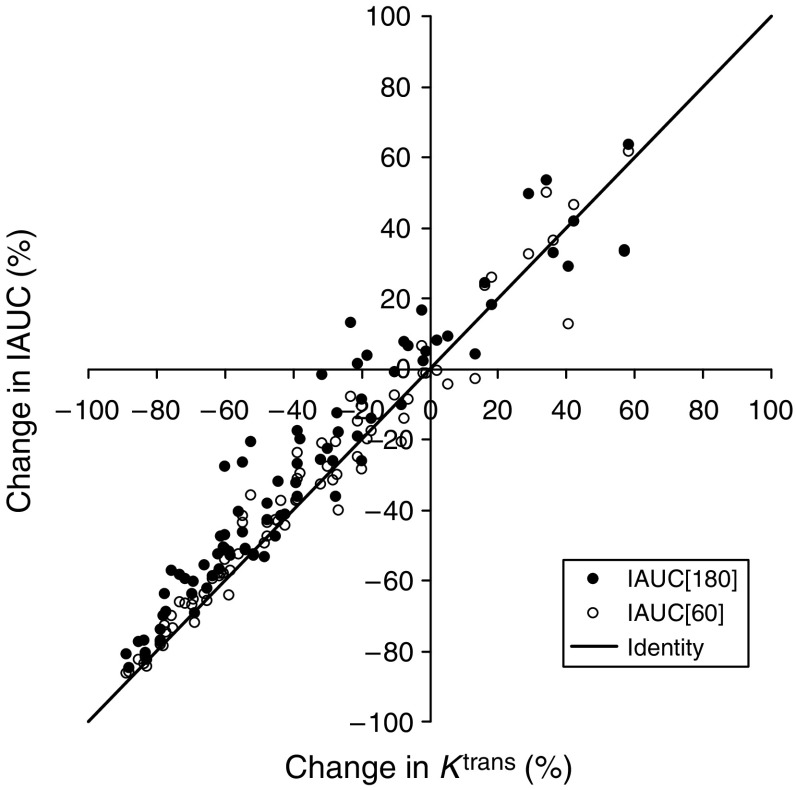
A comparison of the percentage change of the DCE–MRI parameters IAUC and *K*^trans^ after treatment with PTK/ZK, as calculated from *R*_1_ values.

Review of our previous study using DCE–MRI as a biomarker for the effect of antiangiogenesis therapy in advanced cancer (Thomas *et al*, 2005) showed that for 43 recruited patients with advanced cancer, in four out of 39 available patients the DCE–MRI was unsuccessful. In three cases, the ROI could not be accurately followed through the image series (all these tumours were 3 cm or smaller) and in the other case the imaging slice was planned using a breath hold image rather than during gentle respiration, causing misregistration of a 3.1-cm tumour. For the analysed cases, the difference in *K*^trans^ correlated with the pretreatment *K*^trans^ value, but, similar to this study, there was no correlation of the percentage difference to the pre-treatment *K*^trans^ value. There was good correlation between percent changes on treatment seen in IAUC and *K*^trans^ (Figure 6) calculated from *R*_1_ values. After 1 day (3 doses) of treatment with PTK/ZK, there were similar significant reductions in *K*^trans^ of 46±6.3% (mean±s.e. of the mean (s.e.m.)) and IAUC(60) of 43.5±6.5%. For colorectal liver metastases receiving 1000 mg or more of PTK/ZK per day (*n*=14), the effect was increased with a reduction in *K*^trans^ of 55.8±5.6% and IAUC(60) of 55.1±4.9%, again both showing a similar treatment effect.

## DISCUSSION

Dynamic contrast-enhanced MR imaging using an inversion recovery snapshot FLASH sequence with a relatively long *TI*_eff_ of 815 ms and an interval of 3 s between subsequent images, performed in a coronal oblique plane provides a rapid, reasonably accurate measure of *R*_1_ for values between 1 and 5 s^−1^, and therefore of Gd-DTPA concentration during contrast enhancement. The *R*_1_ values seen in the clinical arm of this study were within this range. As *S*_0_ is not continuously measured as *R*_1_ changes, there are errors of up to 6% in the estimated *R*_1_. Systematic errors also occur in estimating dynamic parameters based on signal intensity, as MRI signal intensity is not proportional to Gd-DTPA concentration. These errors do not necessarily affect the reproducibility of contrast enhancement parameters but may affect the assessment of change in enhancement owing to treatment.

Using a long TI_eff_ is helpful as it provides good contrast to noise for low levels of enhancement such as those that may occur in necrotic tumours. However, this has the disadvantage that arterial contrast concentration cannot be accurately measured owing to rapid relaxation during the long TI_eff_ period. Sequences designed to measure blood concentration of contrast agent (Fritz-Hansen *et al*, 1998) have a very short TI_eff_ (150 ms), which may show little or no enhancement in necrotic tumours as they often have a relatively short precontrast *R*_1_.

Measurement of arterial Gd-DTPA concentration (AIF) is considered important when assessing absolute values of tumour microcirculation (Evelhoch, 1999). However, the AIF may not be so important for measuring a treatment effect in patients, unless there is a change in cardiac function or haemodynamics on therapy. Techniques that allow accurate, reproducible measurement of the AIF as well as tissue enhancement may well improve the reproducibility of these parameters, but this study shows that suboptimal measurement of AIF makes the reproducibility of the study worse than if a standardised AIF is used.

The simplified equation (equation (2)) used to convert signal intensities into *R*_1_ values does not take into account the signal saturation due to successive inversion pulses. If the TR_0_ were to be shortened, the estimated *R*_1_ would become progressively more inaccurate. Imaging of tumours with a short precontrast *R*_1_ can be a problem with this sequence as in some cases enhancement may be negative in the first few images of the dynamic series. In a DCE–MRI study, rectified signal can be recognised if the signal reduces in the first few images of contrast enhancement. In practice, this is rarely a problem in liver metastases and this correction was not required in the clinical arm of this study as precontrast *R*_1_ was always greater than 0.53 s^−1^. This problem could be overcome by using saturation recovery rather than inversion recovery, at the expense of lower contrast at shorter *R*_1_ values.

Although respiratory motion causes the tumour to move throughout the series of image acquisitions, the use of the coronal oblique plane minimises through plane motion and repositioning the ROI for each image accounts for in-plane motion. The fact that there was no need to change the size or shape of the ROI suggests that the effect of through plane motion was indeed small. The use of a single region of interest for the whole tumour does not allow discrimination of regions within the tumour, but it does give a good signal-to-noise ratio by averaging the signal from a large volume of tissue and minimises partial volume effects. Acquiring multiple slices, or three-dimensional images, through the tumour and then averaging the tumour ROI for each slice would allow even better signal-to-noise but this would reduce the temporal resolution of the sequence.

The CoV of the repeated measures depends on the preprocessing (use of raw signal intensities or conversion to *R*_1_), analysis method (peak enhancement, IAUC or *K*^trans^) and patient cohort used. Peak enhancement is a simple measure, shown to have similar reproducibility to IAUC. However, peak enhancement is much more a reflection of the extravascular, extracellular space volume fraction (*v*_e_) rather than *K*^trans^, as changes in *K*^trans^ shift the position of the peak of the enhancement curve more than its amplitude, while peak enhancement is, in the limit of large *K*^trans^, proportional to *v*_e_. The validity of using peak enhancement as a biomarker for angiogenesis would therefore seem questionable.

The study shows it is important to use some method for ‘standardising’ the signal intensities from the dynamic image series. The results using calculated *R*_1_ values for IAUC(60) and IAUC(180) and *R*_1_ values with a standard AIF for *K*^trans^, show a CoV ranging from 11 to 19.1%. Assuming the post-treatment CoV is similar, this value can be used to assess the statistical power of studies or anticipate the patient numbers required for a study. Interestingly, our previous published data (Morgan *et al*, 2003) show a 58% mean reduction of *K*_i_ (equivalent to *K*^trans^) with an s.e.m. of 5.2% for 15 patients with colorectal liver metastases treated with PTK/ZK with 1000 mg or more. This is consistent with a CoV of 20% (the CoV is equivalent to the standard deviation (s.d.) of percent changes and the s.e.m. is the s.d. divided by square root of the number of cases). Reanalysis of our other published work (Thomas *et al*, 2005) for patients with liver metastases treated with PTK/ZK with 1000 mg or more (*n*=14) showed a 56% mean reduction in *K*^trans^ and a CoV of 21.1%. A CoV of ∼20%, for both the reproducibility study and treatment effect, implies a cohort of 10 patients would be expected to show a 25% treatment effect with statistical significance (power 0.8). If the CoV is reduced to 14%, as for IAUC(60) in tumours greater than 3 cm diameter, then an 18% treatment effect would show significance. Conversely, if a 40% treatment effect is expected then only 3–4 patients would be required to expect a statistically significant result to *P*<0.05.

Evelhoch *et al* (2004) measured the median IAUC parameter (similar to our IAUC(60)) in 19 human tumours with a 7.2-s image acquisition time. They demonstrated pretreatment tumour interpatient CoV of 64% and intrapatient CoV, in repeated measurements without treatment, of 18%, similar to this study. The high CoV for interpatient tumour measurement supports the notion of assessing percentage rather than absolute changes. Galbraith *et al* (2002) assessed reproducibility in 16 patients with tumours 3 cm in diameter or greater. They use an 11 s image acquisition time. Their data are presented in a slightly different manner and uses both pixel-by-pixel and ROI analysis. For ROI analysis, the data can be summarised to show that for a cohort of 16 patients, IAUC can measure greater than 12% changes and *K*^trans^ can measure 14–17% changes. Similarly, our data extrapolated for 16 patients and tumours 3 cm or greater, (IAUC(60) CoV=14% and *K*^trans^ CoV=16%) would be sensitive to 14 and 16% changes, respectively. Both studies use similar methodology and do not measure AIF, but our study has an image acquisition time of less than 500 ms as opposed to 7.2 and 11 s, dropping the requirement for multiple breath holds and increasing temporal resolution, but at the expense of signal to noise of any given image.

The repeatability varied from 26.5% for IAUC(60) (tumours of diameter greater than 3 cm) to 36.1% for *K*^trans^ (whole group). This is a measure of the significance of an individual result. From our previously published data (Morgan *et al*, 2003; Thomas *et al*, 2005), a 40% change in enhancement parameters is considered to be clinically significant (the change required to predict a tumour response in colorectal liver metastases). A 40% change in an individual patient can therefore be considered both a statistically and a clinically significant finding. Both *K*^trans^ and IAUC are shown to give similar results in the clinical application of this technique and the improved reproducibility of IAUC in this study suggests it is a valuable, straightforward method of evaluating contrast dynamics from DCE–MRI.

In this study, DCE–MRI failed in one patient owing to incorrect positioning of the slice. The incorrect placement was demonstrated by studying the reference slice on both *T*_1_- and *T*_2_-weighted images but was more apparent on *T*_2_-weighted imaging as central tumour necrosis could be seen. When selecting the target lesion, we suggest avoiding metastases with very high *T*_2_-weighted signal intensity to avoid purely necrotic/cystic tumours and to select metastatic deposits with a diameter of greater than 3 cm.

In summary, this technique provides a rapid, straightforward, robust method of measuring tumour enhancement to monitor therapy. All stages of analysis are simple to perform if equation (2) is used to calculate *R*_1_ and IAUC is used to assess tumour enhancement. The speed of image acquisition freezes motion, allowing a wide variety of tumour applications. Also, as multiple breath holds are not required, the scanning protocol is easier both for patients and scanning technicians.

## Figures and Tables

**Table 1:** Summary of parameters derived from two DCE–MRI scans, performed 1 week apart, without therapeutic intervention

**Parameter**	**Mean change (%)**	**CoV (%)**	**Repeatability (%)**
*Unscaled signal intensity data*			
PE	21.2	48.6	99.5
IAUC(60)	15.4	50.1	98.0
IAUC(180)	17.0	50.0	98.7
			
*Signal intensity related to measured arterial input function*			
PE/IAUC-A(180)	12.0	35.7	70.4
IAUC(60)/IAUC-A(60)	6.6	37.2	70.3
IAUC(180)/IAUC-A(180)	8.3	37.8	72.1
*K*^trans^	4.6	34	75.0
			
*R*_*1*_ *data*			
PE	1.0	15.9	29.5
IAUC(60)	1.7	15.8	29.5
IAUC(180)	0.9	16.1	29.9
*K*^trans^	3.1	19.1	36.1

PE=peak enhancement; IAUC(*t*)=initial area under the *tumour* contrast enhancement curve for first *t* seconds; IAUC-A(*t*)=Initial area under the *arterial* contrast enhancement curve for first *t* seconds.

**Table 2:** Individual patient data showing tumour size, mean difference, coefficient of variation (CoV) and repeatability for *K*^trans^ and IAUC(60) for two scans, 1 week apart, without therapeutic intervention

			***K*^trans^ (min^−1^)**			**IAUC(60)**		
**Site**	**Primary**	**Size (cm)**	**Scan 1**	**Scan 2**	**% change**	**Scan 1**	**Scan 2**	**% change**
Liver	Colorectal	7	0.116	0.111	−4.3	10.1	9.7	−3.7
Liver	Colorectal	2.8	0.018	0.014	−22.2	1.2	1.1	−15.2
Liver	Colorectal	4.7	0.116	0.100	−14.0	9.3	8.2	−11.6
Liver	Colorectal	6	0.186	0.192	3.2	16.6	15.1	−9.1
Liver	Colorectal	10.4	0.037	0.044	18.9	3.6	4.2	15.6
Liver	Colorectal	17	0.085	0.081	−4.7	6.7	6.7	−0.5
Liver	Lung	4.6	0.039	0.051	30.8	3.5	4.3	24.1
Lung	Lung	2.5	0.231	0.298	29.0	17.4	21.0	20.8
Lung	Lung	7	0.152	0.171	12.5	12.6	14.5	15.0
Lymph node	Melanoma	2.4	0.49	0.40	−18.5	35.0	28.7	−18.0
								
			***K* ^trans^**		**IAUC(60)**			
All cases	Mean change (%)		3.1		1.7			
	CoV (%)		19.1		15.8			
	Repeatability (%)		36.1		29.5			
Size>3 cm	Mean change (%)		6.1		4.3			
	CoV (%)		15.5		13.9			
	Repeatability (%)		30.6		26.5			

IUAC=initial area under the contrast enhancement curve.

Parameters were calculated using *R*_1_ values and a standardised arterial input function for *K*^trans^.
